# Mechanisms and Advances in the Treatment of Arrhythmias Associated With Pulmonary Hypertension

**DOI:** 10.31083/RCM52744

**Published:** 2026-09-04

**Authors:** Caijuan Huan, Zhaoming Fu, Rong Jiang, Shuo Liu, Xuan Zhang, Jian Yang

**Affiliations:** ^1^Department of Respiratory Medicine, The First Affiliated Hospital, College of Medicine, Zhejiang University, 310003 Hangzhou, Zhejiang, China; ^2^Department of Cardiology, The First Affiliated Hospital, Zhejiang University School of Medicine, 310003 Hangzhou, Zhejiang, China

**Keywords:** pulmonary hypertension, arrhythmias, electrical remodeling, structural remodeling, heart failure

## Abstract

Arrhythmias related to pulmonary hypertension (PH) are major complications of disease progression and are associated with a significantly increased risk of death. The pathophysiology of these arrhythmias involves complex, multifaceted mechanisms. Arrhythmic substrates include electrical remodeling (ion channel imbalance, gap junction protein connexin 43 (Cx43) dysfunction, and autonomic neuropathy), structural remodeling (right ventricular hypertrophy/fibrosis), metabolic disorders (mitochondrial dysfunction and the Warburg effect), and chronic inflammation. Clinically, PH-related atrial arrhythmias, particularly atrial fibrillation (AF)/flutter (AFL), are the most common. Diagnosis often requires combining ambulatory electrocardiography and implantable cardiac monitoring to detect subclinical events. Current treatment methods include optimizing PH-targeted therapy (e.g., dual- or triple-targeted drug therapy, soluble guanylate cyclase (sGC) stimulators), antiarrhythmic drugs, and catheter ablation, as well as emerging therapies such as Cx43 modulators, gene therapy (targeting bone morphogenetic protein receptor type 2 (*BMPR2*)/*switch-independent 3A* (*SIN3A*)), and microRNA (miRNA)-based regulation. Future work should focus on elucidating underlying mechanisms, developing precise risk stratification models, and formulating targeted individualized intervention strategies.

## 1. Introduction

Pulmonary hypertension (PH) is a syndrome with diverse causes, characterized by pulmonary vascular remodeling and elevated pulmonary vascular resistance (PVR). It leads to right ventricular dysfunction, heart failure, and high mortality. Arrhythmias are common complications that indicate disease progression and hemodynamic instability, and are independent risk factors for mortality [1]. Advances in the hemodynamic definitions, clinical classification, and therapeutic targets of PH make it crucial to understand arrhythmia mechanisms and treatment advances for clinical practice and research.

### 1.1 Definition and Epidemiology of Pulmonary Hypertension

Right heart catheterization (RHC) is the gold standard for PH diagnosis. Traditional criteria defined mean pulmonary arterial pressure (mPAP) as ≥25 mmHg, which may delay early intervention. In 2022, the European Society of Cardiology/European Respiratory Society (ESC/ERS) guidelines updated the criteria to mPAP >20 mmHg [2]. Evidence shows a significantly increased risk of all-cause mortality and cardiovascular hospitalization in the population with mPAP of 21–24 mmHg [3,4]. The guidelines emphasize that pulmonary artery wedge pressure (PAWP) ≤15 mmHg remains the recommended threshold for pre-capillary PH, while PAWP >15 mmHg defines post-capillary PH, serving as the key hemodynamic differentiator between pre- and post-capillary status. Furthermore, among patients already diagnosed with post-capillary PH, PVR >2 WU is a critical parameter for distinguishing combined pre- and post-capillary PH (CpcPH, PVR >2 WU) from isolated post-capillary PH (IpcPH, PVR ≤2 WU) [2]. PH is a global public health issue with an adult prevalence of ≥1%. Rising detection rates are linked to aging, increased awareness, and non-invasive screening technologies [5]. However, regional differences exist: in Europe and the US, Group 2 PH (PH associated with left heart disease, PH-LHD) predominates [5]. The PRO-KERALA study in India found that PH-LHD accounts for 59% of cases, with 42% being type 1 PH, significantly higher than in the West. Additionally, a high proportion of PH cases were linked to congenital heart disease and connective tissue disease, underscoring genetic and environmental factors [6].

### 1.2 Clinical Burden and Prognosis of PH-Associated Arrhythmias

#### 1.2.1 Epidemiology of PH-Associated Arrhythmias

In PH patients, arrhythmias have a high incidence and major clinical impact, with atrial arrhythmias (AA, predominantly atrial fibrillation (AF) and atrial flutter (AFL)) being the most prevalent and extensively studied [7]. AA is associated with elevated right atrial pressure and increased N-terminal pro-B-type natriuretic peptide (NT-proBNP) levels, suggesting its link to hemodynamic instability; however, AA is not merely a marker of disease severity but may act as an independent factor contributing to clinical deterioration [8]. Ventricular arrhythmias (VA) occur less frequently but have serious clinical consequences; they are not the main cause of sudden cardiac death (SCD) in PH patients, whereas AA—particularly AF—is more strongly associated with clinical deterioration and increased mortality [9]. The specific epidemiological profiles, diagnostic limitations, and prognostic implications of AA will be elaborated in Section 3.1.1.

#### 1.2.2 Prognosis of PH-Associated Arrhythmias

Paroxysmal atrial arrhythmias indicate a poor prognosis in PH patients. De novo atrial arrhythmias nearly quadruple the death risk, independent of other risk factors [10]. PH patients with de novo AFL/AF have a 5-year survival rate of only 47%, 21 percentage points lower than those without atrial arrhythmias (68%), and sinus rhythm restoration can improve survival [11]. PH patients depend on the atrial kick to sustain cardiac output, which declines sharply during atrial arrhythmias. 97.5% of patients experience clinical deterioration, making sinus rhythm restoration a core treatment goal [12].

### 1.3 Aims of the Study

Given the complexity and clinical significance of PH-related arrhythmias and current treatment limitations, this review integrates recent advances, evaluates existing treatments, examines emerging technologies, and identifies knowledge gaps. It aims to guide research, provide clinical recommendations, promote personalized interdisciplinary models, and improve patient quality of life and prognosis.

## 2. Mechanisms of PH-Related Arrhythmias

PH-induced arrhythmia involves a complex pathophysiological network with multiple intertwined factors. Its core mechanisms encompass three major dimensions: electrophysiological remodeling; structural remodeling; and inflammatory activation and immune imbalance. These interact, forming a microenvironment that promotes arrhythmia occurrence and maintenance.

### 2.1 Electrical Remodeling: Imbalance in Myocardial Electrical Activity

#### 2.1.1 Ion Channel Dysfunction and Abnormal Action Potentials

Precise regulation of the action potential (AP) is essential for cardiac synchronous contraction. The right ventricle (RV) serves as the principal site of electrical remodeling in PH. Conduction disturbances, repolarization abnormalities, and electromechanical dyssynchrony are markedly more pronounced in the RV compared with the left ventricle (LV). These alterations constitute the fundamental electrophysiological substrate for arrhythmogenesis and the progression of cardiac dysfunction [13]. In PH-induced right ventricular pressure overload, ion channel remodeling disrupts myocardial electrical stability [14,15]. Notably, dynamic imbalances in K^+^ and Ca^2+^ channels characterize this process: during the compensated right ventricular (RV-C) phase, electrical remodeling remains subtle.

In the decompensated right ventricle (RV-D) phase, downregulation of key voltage-gated potassium (K_V_) channel subtypes (e.g., K_V_1.2, K_V_1.5, K_V_4.3) causes repolarization delay, action potential duration (APD) prolongation, and reduced repolarization reserve, increasing the risk of early afterdepolarizations (EADs) and ventricular arrhythmias. Abnormal inward rectifier potassium (Kir) channel function destabilizes the resting potential, further exacerbating electrical dispersion. Voltage-dependent calcium channel (Ca_V_)1.2/*CACNA2D1* downregulation reduces calcium influx, impairs excitation–contraction coupling, promotes arrhythmogenic Ca^2+^ release, and causes electrical instability. Essentially, RV-D remodeling involves potassium/calcium channel dysregulation, leading to APD prolongation, resting-potential instability, and disrupted Ca^2+^ handling, thus providing a substrate for malignant arrhythmias [13,16]. Sodium channel inhibition is another key mechanism in PH–related right heart failure electrical remodeling: reduced Na_V_1.5 expression and attenuated INa impair AP depolarization and conduction while prolonging APD. This downregulation co-occurs with K_V_/K_ir_ channel imbalance, further extending APD and the QT interval and increasing EAD risk. Additionally, decreased expression of Na_V_1.5, desmoglein-2, and connexin 43 (Cx43) disrupts intercellular electrical coupling, promoting conduction heterogeneity and ventricular arrhythmias. Together, sodium channel inhibition and potassium channel dysfunction prolong APD and may disrupt calcium homeostasis via the sodium-calcium exchanger (NCX), forming a pro-arrhythmic network central to PH-related right heart failure electrical remodeling [16]. Of note, right ventricular ischemia secondary to PH is exceedingly rare. PH also impairs sinoatrial node function: monocrotaline (MCT)-induced PH rat models show decreased intrinsic heart rate and automaticity, evidenced by a reduced diastolic depolarization slope and APD prolongation. Molecular remodeling involves ion channel dysregulation (e.g., hyperpolarization-activated cyclic nucleotide-gated (HCN)1/HCN4, Ca_V_1.2/Ca_V_1.3/Ca_V_3.1 downregulation, K_V_1.4*/*K_V_4.2 inhibition), calcium homeostasis imbalance (e.g., NCX1, sarcoplasmic/endoplasmic reticulum Ca^2+^-ATPase 2 (SERCA2), ryanodine receptor 2 (RyR2) downregulation disrupting the calcium clock), and interstitial fibrosis (e.g., vimentin, collagen, transforming growth factor-beta (TGF-β)1 upregulation). *TBX3 *downregulation correlates with ion channel remodeling and weakened pacing ability, which coincides with PH mortality, and suggests that sinoatrial node dysfunction may drive fatal bradyarrhythmias in PH [17]. Temple et al. [18] found that PH downregulates the atrioventricular node (AVN) ion channel transcriptome, reducing L-type Ca^2+^ channels Ca_V_1.2/Ca_V_1.3 and HCN channel expression; this impairs conduction, prolongs the refractory period, and provides a molecular basis for re-entrant arrhythmias.

#### 2.1.2 Gap Junction Remodeling and Conduction Disorders

Gap junctions, composed of connexin proteins (Cx), are crucial for electrical impulse conduction between myocardial cells. Their dysfunction and aberrant distribution can trigger conduction slowing, conduction block, and reentrant arrhythmias. Cx43, the most abundant gap junction protein in humans, is widely distributed across tissues and forms intercellular channels that facilitate electrical signaling and mechanical synchronization [19]. In PH-induced right ventricular pressure overload, myocardial fibrosis and an inflammatory microenvironment disrupt Cx43 homeostasis. Cx43 absence, altered phosphorylation, and lateralization impair electrical coupling and synchrony, reduce conduction velocity, and create heterogeneous conduction anisotropy, thereby promoting lethal reentrant circuits [20]. MicroRNAs (miRNAs) regulate PH by inhibiting gene expression through binding to mRNA 3′ untranslated regions (UTRs). Their aberrant expression in PH patients plays a key role in regulating cellular proliferation, apoptosis, and metabolism [21]. For example, miR-17/92 exacerbates PH by suppressing the bone morphogenetic protein receptor type 2 (*BMPR2*) pathway and reducing apoptosis in pulmonary artery smooth muscle cells (PASMCs) [22]; miRNAs also regulate signal transducer and activator of transcription 3 (STAT3) signaling during PH pathogenesis [23]. Although current PH research predominantly focuses on vascular remodeling and pressure elevation, miRNA dysregulation may also influence cardiac electrical activity by modulating ion channels and gene expression [24]. Future studies should investigate the role of miRNAs in PH-associated arrhythmias to identify novel therapeutic avenues; however, current evidence derives largely from animal models and lacks validation in humans. In the PH myocardium, impaired energy metabolism and oxidative stress can induce pathological opening of Cx43 hemichannels, leading to leakage of small molecules such as ATP and excessive Na^+^ influx. This exacerbates intracellular Na^+^ overload, a strong predictor of ventricular fibrillation [25].

#### 2.1.3 Autonomic Nervous System Dysfunction

Chronic hypoxemia and cardiac dysfunction in PH patients reflexively activate the sympathetic nervous system to maintain cardiac output and blood pressure, but sustained sympathetic overactivation induces arrhythmias. Increased sympathetic tone and β-adrenergic receptors (β-AR) signaling: PH patients exhibit enhanced sympathetic activity, upregulating β_1_-AR expression and sensitivity in the right atrium. Persistent activation further increases innervation and β_1_-AR density, enhancing atrial pacemaker cell excitability and inducing atrial arrhythmias through trigger activity and delayed afterdepolarization mechanisms [26]. Renal-cardiac reflex axis: Animal experiments confirm that sympathetic impulses from the left kidney significantly increase AF/AFL susceptibility in PH model animals by stimulating the left stellate ganglion and the anterior right ganglionated plexi (ARGP), revealing the key role of this reflex axis in PH-related atrial arrhythmias [27].

### 2.2 Structural Remodeling: Anatomical Matrix of Arrhythmias

Electrophysiological remodeling sparks arrhythmias, while structural remodeling serves as the ready-made kindling. Chronic pulmonary artery pressure overload drives adverse right ventricular structural remodeling, establishing a persistent anatomical substrate for abnormal electrical conduction.

#### 2.2.1 Right Ventricular Hypertrophy and Dilatation

Initially, in PH, right ventricular afterload increases, and myocytes boost contractility through hypertrophy to adapt [28]. However, when pressure load exceeds compensatory capacity, the RV transitions from concentric to eccentric hypertrophy, characterized by chamber dilation. According to Laplace’s law, dilation alters wall tension, increasing myocardial oxygen consumption; it also promotes tricuspid annular dilation and regurgitation, further raising volume load and forming a vicious cycle [29].

#### 2.2.2 Myocardial Fibrosis

Interstitial fibrosis (diffuse or focal) is a key structural basis for right ventricular failure, and its dysregulation can exacerbate the disease [30]. In PH, activated fibroblasts secrete excessive collagen, which compresses and interferes with myocardial cells’ structure and function. The collagen scar tissue disrupts electrical coupling between cells, surrounds and divides myocardial bundles, forcing impulse waves to detour and slowing conduction velocity. The resulting network creates conduction anisotropy, providing optimal conditions for micro- or macro-reentry [31].

#### 2.2.3 Pathophysiology of Coronary Insufficiency and Arrhythmogenesis

Myocardial hypertrophy increases oxygen demand while supply remains insufficient: elevated right ventricular pressure reduces coronary perfusion pressure, decreasing myocardial perfusion; simultaneously, hypertrophy involves inadequate angiogenesis and capillary rarefaction, reducing capillary density per unit myocardium [27]. This oxygen supply-demand imbalance causes prolonged relative ischemia in the right ventricular myocardium. Ischemia directly impairs cardiomyocyte function and, through local metabolic derangement, alters resting membrane potential and action potential morphology, increasing refractory period dispersion and triggering arrhythmias [32]. Furthermore, PH induces dilation of the main pulmonary artery, thereby compressing the left main coronary artery (LMCA). This compression results in significant LMCA stenosis (typically ≥50%), which leads to left ventricular myocardial ischemia. Following the onset of ischemia due to LMCA compression, the electrophysiological stability of the affected myocardium is compromised: alterations in action potential duration and increased dispersion of repolarization facilitate the initiation of reentrant circuits or triggered activity, ultimately precipitating malignant ventricular tachyarrhythmias, such as ventricular tachycardia and ventricular fibrillation. This pathophysiological mechanism involves the LV as the ischemic region, which is associated with more severe clinical outcomes and often manifests directly as sudden cardiac death [33,34].

#### 2.2.4 Metabolic Reprogramming

In the hyperloaded RV, despite sufficient oxygen, a tumor-like Warburg effect occurs, shifting energy metabolism from fatty acid oxidation to glycolysis [28,35]. This metabolic reprogramming causes mitochondrial dysfunction, leading to overproduction of reactive oxygen species (ROS), heightened oxidative stress, and disrupted Ca^2+^ regulation, which impair membrane potential and contractility. These dysfunctions further promote ROS generation, activate Ca^2+^/calmodulin-dependent protein kinase II (CaMKII), increase Ca^2+^ leakage, and induce pro-arrhythmic effects [36,37]. Inhibition of fatty acid oxidation causes toxic lipid intermediate accumulation, inducing apoptosis and electrophysiological abnormalities [28].

#### 2.2.5 Activation of the Neuroendocrine System

The renin-angiotensin-aldosterone system (RAAS) is activated in PH/right heart failure. Angiotensin II (Ang II) and aldosterone act as vasoconstrictors, promote water and sodium retention, and are profibrotic and proinflammatory. Ang II induces cardiomyocyte hypertrophy, fibroblast proliferation, and ROS generation via receptors. Aldosterone promotes cardiac fibrosis through mineralocorticoid receptors, causing collagen deposition and electrolyte imbalance, which worsens myocardial electrophysiology. These changes collectively increase arrhythmia risk [38,39].

### 2.3 Inflammatory Activation and Immune Imbalance: Multidimensional Drivers of Arrhythmias

Chronic inflammation drives PH through perivascular inflammatory cell infiltration and cytokine network activation, affecting both pulmonary vessels and the RV, and may indirectly promote arrhythmias via myocardial structural remodeling [40].

#### 2.3.1 Proinflammatory Cytokines and Signaling Pathways

PH patients have elevated pro-inflammatory cytokines (e.g., IL-6, TNF-α, IL-1) in circulation [41,42]. Cytokines activate inflammatory pathways (e.g., NF‐κB and MAPK), causing pulmonary vascular inflammation and remodeling [43]. This is the key mechanism of PH progression and offers targets for intervention and treatment [44,45,46]. They significantly contribute to arrhythmias [47,48].

#### 2.3.2 Direct Electrophysiological Effects

Inflammatory cytokines (e.g., TNF-α, IL-1β, IL-6) prolong APD via direct effects on myocardial ion channels, inhibiting IKr and modulating ICaL [47,49].

#### 2.3.3 Indirect Structural Remodeling Effects

NF-κB signaling plays a pivotal role in pressure overload-induced atrial fibrosis and electrical remodeling [50]. In PH models, NF‐κB activation correlates with pulmonary artery pressure and vascular remodeling [51]. Although quantitative data directly linking NF‐κB phosphorylation in ventricular myocytes to ventricular arrhythmias remain lacking, these pathways can plausibly promote myocardial fibrosis and thereby create a substrate for reentry.

#### 2.3.4 Autonomic Nervous System Dysregulation

Inflammation is linked to autonomic nervous system (ANS) imbalance. In experimental PH, it induces oxidative stress, leading to sympathetic overactivity and reduced vagal tone (ANS imbalance), thereby promoting right ventricular dysfunction [52]. Sympathetic overactivation increases arrhythmia risk through electrophysiological disturbances, myocardial remodeling, and exacerbated inflammation, whereas parasympathetic hypofunction diminishes its protective effects [39].

In summary, the mechanism of PH-related arrhythmias involves multiple factors—abnormal ion channels, structural changes in myocardial tissue, metabolic disorders, and neuro-endocrine-immune imbalances—collectively increasing arrhythmia risk in the right atrium. Future research should investigate interactions between these mechanisms, such as metabolic effects on ion channels or inflammatory impacts on gap junctions, to facilitate multi-targeted therapies.

## 3. Clinical Characteristics and Diagnostic Strategies for PH-Related Arrhythmias

### 3.1 Clinical Features and Diagnosis of PH-Related Arrhythmias

#### 3.1.1 Atrial Arrhythmias

Atrial arrhythmias, such as AF, AFL, and other supraventricular tachycardias, are common in PH patients, linked to right atrial enlargement and indicating disease severity. A large Chinese PH study found that 44.4% of patients had at least one arrhythmia, with supraventricular arrhythmias at 18.5%. Among these, AF prevalence was 8.1%, AFL was 4.1%, and other atrial tachycardias were 10.2%. AF incidence in PH is significantly higher than in the general population [53]. Notably, the underlying etiology of PH influences the pattern of atrial arrhythmia presentation [54].

In the past, patients with poorly controlled PH often died prematurely, precluding the development of age-related comorbidities. With the widespread use of prostacyclin analogues and other advanced therapies, however, survival has been substantially prolonged, and an increasing number of PH patients now reach middle or old age. Consequently, they become susceptible to atherosclerotic cardiovascular disease and ischemic cardiomyopathy, which may serve as important triggers for PH-related atrial arrhythmias. On the one hand, advancing age is an established independent risk factor for atrial arrhythmias [2]. On the other hand, ischemic cardiomyopathy arising from left-sided heart disease can lead to left ventricular dysfunction and elevated left atrial pressure, which in turn aggravates PH and markedly increases the incidence of AF, thereby creating a vicious cycle. However, a direct causal linkage whereby ischemic cardiomyopathy may indirectly precipitate AF through the induction of PH remains to be empirically validated [55]. In patients with PH-LHD, AF (rather than all atrial arrhythmias) is significantly associated with CpcPH, and left atrial enlargement is an important mediator of this association [56]. Nevertheless, a paucity of robust evidence persists in current investigations examining the association between CpcPH and atrial arrhythmias. Conventional electrocardiogram (ECG) often fails to accurately reflect atrial arrhythmia burden, particularly in paroxysmal and asymptomatic cases. Holter monitoring studies have shown that nearly one-third of PH or chronic thromboembolic pulmonary hypertension (CTEPH) patients have undiagnosed paroxysmal AF, suggesting that the actual burden is higher than clinically expected; thus, extended cardiac monitoring is recommended for high-risk PH patients [26]. Atrial arrhythmias harm PH patients’ hemodynamics and prognosis, with AF/AFL onset or persistence linked to clinical deterioration, worsened right heart failure, and increased mortality [54]. This harm manifests in two main aspects. The first is hemodynamic deterioration caused by the rapid ventricular rate, which impairs ventricular filling and attenuates atrial contraction. This consequently reduces cardiac output and aggravates tricuspid regurgitation [54]. The second is an elevated thromboembolic risk, driven by PH-induced blood stasis, endothelial injury, and hypercoagulability. These factors increase the risk of intracardiac thrombosis in AF patients, potentially leading to ischemic stroke and an increased risk of hemorrhagic stroke, both of which contribute to a poor prognosis [57].

#### 3.1.2 Ventricular Arrhythmias

PH patients have a lower incidence of ventricular arrhythmias than atrial arrhythmias [14,53], but they may signal clinical deterioration [58]. Data from Chinese PH patients show an overall ventricular tachycardia (VT) prevalence of 2.5% (including non-sustained VT). Due to monitoring limitations, asymptomatic VT episodes may be underrecorded, leading to underestimation of their significance, and further research is needed for verification [53]. Among patients with intermediate-high and high risk acute pulmonary embolism (PE), the incidence of prolonged corrected QT interval (QTc) (defined as ≥470 ms in males and ≥480 ms in females) on admission was 28.4%, significantly exceeding the previously reported range of 10%–20%. This prolonged QTc interval was associated with elevated lactate levels at admission. In contrast, no significant differences were observed in right ventricular function parameters assessed by echocardiography or computed tomography between patients with normal and prolonged QTc intervals, indicating that the prolonged QTc interval may reflect systemic hypoxia and myocardial metabolic stress rather than isolated right ventricular structural abnormalities. Notably, irrespective of whether anticoagulation, thrombolysis, or interventional therapy was administered, the QTc interval normalized prior to discharge and was not associated with increased in-hospital or long-term mortality. No malignant arrhythmias, including torsades de pointes, were observed, confirming that this represents an acute and reversible repolarization abnormality [59].

#### 3.1.3 Bradycardia

Bradycardia (primarily due to sinus node dysfunction [SND] or atrioventricular block [AVB]) is less common than tachycardia in PH but can significantly compromise hemodynamics. Reported prevalence in PH patients varies: a 2025 European prospective study reported approximately 5%–10%, mostly transient and self-limiting, with 6% (19/314) requiring intervention or becoming symptomatic [60]. A large retrospective study in a Chinese PH population found a SND prevalence of 12.2% and an AVB of 7.1% [53]. These discrepancies likely reflect baseline differences across study populations (age, comorbidities, and PH aetiological mix), which current evidence identifies as the main driver. In PH, heart rate compensates for the reduced right ventricular stroke volume imposed by elevated afterload, thereby preserving cardiac output. This compensation, however, is fragile—even brief asymptomatic sinus pauses or high-grade AV block can precipitously lower cardiac output, producing dizziness and fatigue, or triggering further arrhythmias [61].

#### 3.1.4 AF/AFL in Adult Congenital Heart Disease Patients With PH

Patients with congenital heart disease (CHD) demonstrate a substantially elevated incidence of AF/AFL, along with an earlier age of onset, relative to the general population. This disparity is primarily attributable to persistent hemodynamic disturbances, including pressure or volume overload resulting from intracardiac shunts, PH, and prior surgical interventions. PH promotes atrial dilation, fibrosis, and conduction slowing, thereby establishing an arrhythmogenic substrate. Even in patients with simple forms of CHD, such as atrial septal defect or ventricular septal defect, chronic shunting drives progressive atrial remodeling, ultimately elevating susceptibility to atrial arrhythmias. The present study further indicates that hemodynamic determinants—specifically PH and CHD severity—constitute more significant predictors of arrhythmia risk in this population than traditional metabolic factors, such as hypertension and diabetes. Notably, both the age of onset and the predominant arrhythmia phenotypes vary across distinct CHD subtypes. Consequently, the arrhythmogenic mechanisms in CHD are predominantly driven by hemodynamic factors (especially PH and severity), distinguishing them markedly from those in the general population [62].

### 3.2 Diagnostic and Monitoring Strategies: From Basic to Cutting-Edge

#### 3.2.1 Standard 12-Lead ECG

ECG is a key tool for initial and follow-up assessment in PH, providing data on right heart strain (e.g., P pulmonale, right bundle branch block, right axis deviation, RV hypertrophy and strain) to predict arrhythmia risk and prognosis. However, it should be combined with clinical manifestations and other tests for comprehensive judgment [63].

#### 3.2.2 Ambulatory ECG (Holter) Monitoring

For patients suspected of paroxysmal arrhythmias (e.g., paroxysmal AF, non-sustained supraventricular tachycardia) or with unexplained palpitations or syncope, 24- to 48-hour Holter monitoring is a key method. It improves detection of occult (especially paroxysmal) arrhythmias and compensates for routine ECG limitations [26].

#### 3.2.3 Implantable Loop Recorder

The implantable loop recorder (ILR) offers continuous cardiac monitoring for years in PH patients, improving risk stratification and prognosis prediction via heart rate variability and activity analysis. It supplements the ESC/ERS guidelines and has evolved from passive symptom tracking to active assessment of disease progression and risk [64].

#### 3.2.4 Invasive Electrophysiological Study

The use of electrophysiological study (EPS) in PH patients remains limited (around 2.2%), primarily indicated for arrhythmias like non-sustained ventricular tachycardia (nsVT), AFL, and atrioventricular nodal reentrant tachycardia (AVNRT) that require mechanism clarification or ablation. EPS is proven effective for atrial arrhythmias (especially AFL and AVNRT), with ablation success rates similar to those of non-PH patients. However, for ventricular arrhythmias, though nsVT is common, EPS rarely induces sustained VT/ventricular fibrillation (VF), and its prognostic value remains uncertain. Thus, EPS should be strictly indicated, mainly for diagnosing and treating atrial arrhythmias, while ventricular arrhythmia risk assessment should be cautious and clinical context-dependent [65].

#### 3.2.5 Wearable Technology

Consumer-grade wearable devices demonstrate considerable potential for the diagnosis and monitoring of arrhythmias and for the assessment of physical activity in PH. In the field of arrhythmia, applications predominantly focus on the screening and detection of AF. Contemporary smartwatches and wristbands utilize photoplethysmography (PPG) and ECG sensors to achieve AF identification with high sensitivity and specificity (ranging from 91% to 100%). Large-scale clinical trials, such as the Apple Heart Study and the China Mobile Atrial Fibrillation Screening Study, have confirmed the positive predictive value of these devices in real-world settings (84% and 91.6%, respectively). Furthermore, they are capable of detecting asymptomatic AF, which may contribute to a reduced risk of stroke. Additionally, smart garments (e.g., Cardioskin) can record continuous 15-lead ECGs, smart glasses can detect AF through facial PPG, and smart rings have integrated heart rate monitoring functionalities, thereby expanding the spectrum of device forms available for arrhythmia monitoring. Regarding PH, wearable devices are primarily employed for the objective assessment of physical activity levels. By measuring daily step count, activity duration, and moderate-to-vigorous physical activity via accelerometers, these metrics demonstrate a favorable correlation with the 6-minute walking distance (6MWD) and quality-of-life questionnaires. However, they are not entirely consistent with traditional hemodynamic parameters, suggesting that fatigue and activity limitation result from comprehensive multi-system effects. Several pharmacological and device trials (e.g., involving inhaled nitric oxide, pulmonary denervation TROPHY1, VENTASTEP, TRACE), have adopted wearable activity monitoring as primary or secondary outcome measures to evaluate the impact of therapeutic interventions on patients’ daily activity capacity, underscoring its value in efficacy evaluation and research support [66]. Nevertheless, there is currently a paucity of research concerning the application of wearable devices for PH-related arrhythmias.

## 4. Treatment Strategies and Emerging Treatments

### 4.1 Treatment for Pulmonary Hypertension

The efficacy of all antiarrhythmic treatments depends on effective control of PH. This is achieved by reducing pulmonary arterial pressure and vascular resistance, alleviating right ventricular load, and reversing or mitigating right atrial and right ventricular dilation, fibrosis, and electrical remodeling—thereby ultimately reducing arrhythmia risk.

#### 4.1.1 Targeted Drug Combination Therapy

Targeted drug combination is central to the treatment of PH. According to the 2022 ESC/ERS guidelines, initial monotherapy is recommended for patients with cardiopulmonary comorbidities, for whom evidence for combination therapy is lacking. For newly diagnosed PH patients without comorbidities who present at low or intermediate risk, initial dual combination therapy with an endothelin receptor antagonist (ERA) and a phosphodiesterase type 5 inhibitor (PDE5i) is recommended [2]. This recommendation is supported by the AMBITION trial, which demonstrated that initial combination therapy with ambrisentan (ERA) and tadalafil (PDE5i) significantly reduced the risk of clinical failure by 50% compared with monotherapy, and significantly improved 6MWD [67]. The 2024 World Symposium on Pulmonary Hypertension (WSPH) further reinforced this paradigm, recommending upfront combination therapy with early and frequent reassessment as the standard of care for most patients with PH [68].

#### 4.1.2 Triple Targeted Therapy

Initial triple therapy improves survival in high-risk idiopathic PH patients unresponsive to nitric oxide (NO) [69]. Triple therapy (ERA + PDE5i + prostacyclin analogue) is the recommended initial regimen for high-risk PH patients; for intermediate-risk patients, initial dual oral therapy (ERA + PDE5i) is preferred, with sequential addition of a prostacyclin pathway agent only if low-risk status is not achieved during follow-up [70,71]. Studies show that sequential triple therapy (adding selexipag to ERA + PDE5i/riociguat) improves long-term survival in PH patients, with 1-year and 2-year rates of 86.5% and 86.0%. It also benefits exercise tolerance, cardiac remodeling, and biomarkers across risk strata [72]. However, more research is needed to verify whether sequential triple therapy is superior to dual therapy. Large-scale randomized controlled trials (RCTs) with arrhythmia endpoints are lacking; theoretically, its advantage in improving right heart function may indirectly optimize arrhythmia management.

#### 4.1.3 Application of sGC Stimulators

Soluble guanylate cyclase (sGC) stimulators, such as riociguat, are key in treating PH by activating sGC, enhancing NO sensitivity, strengthening the NO-sGC-cyclic guanosine monophosphate (cGMP) pathway, and promoting pulmonary vasodilation [73,74]. Riociguat significantly reduces PVR and improves pulmonary artery compliance (PAC), potentially lowering right ventricular workload by reducing pulsatile afterload [74,75].

### 4.2 Antiarrhythmic Drug Therapy

#### 4.2.1 Rate-Control Medications

Rate control is first-line for atrial arrhythmias, particularly AF. Digoxin, a traditional choice, has a narrow therapeutic window and may cause toxicity with renal insufficiency or electrolyte disorders [76]. Beta-blockers are controversial in PH patients due to their negative inotropic effect, which may worsen right heart failure. Although commonly used for left heart failure, only low doses of selective beta1-blockers (e.g., bisoprolol) may be considered in specific cases, such as controlling AF with a rapid ventricular rate, requiring strict hemodynamic monitoring. Non-dihydropyridine calcium channel blockers (e.g., verapamil, diltiazem) should be used cautiously in PH patients with right heart failure due to their significant negative inotropic effect [1,54].

#### 4.2.2 Rhythm Control Medications

The use of antiarrhythmic drugs (AADs) in PH is limited and carries substantial risk. Amiodarone remains the primary agent for rate and rhythm control; however, evidence remains scarce and conflicting, with no large-scale clinical trials of amiodarone or dronedarone specifically in PH patients [14,76]. A critical concern is pulmonary toxicity (manifesting as interstitial pneumonia or pulmonary fibrosis) [77,78], given that PH patients already have severe pulmonary vascular remodeling; even minor lung injury can be fatal, warranting heightened vigilance. In contrast, class I AADs (e.g., flecainide, propafenone) exert prominent negative inotropy and proarrhythmic effects and are therefore contraindicated in structural heart disease, particularly right heart failure [26].

#### 4.2.3 Anticoagulation Therapy

Patients with PH and AF have a high thromboembolism risk due to right atrial enlargement, blood stasis, and hypercoagulability, and the bleeding risk also increases, especially for hemorrhagic stroke. Therefore, long-term anticoagulation should be individually assessed to balance thrombosis and bleeding risks, not blindly follow unified protocols [57]. Warfarin is the traditional choice but requires regular monitoring. Direct oral anticoagulants (DOACs) are increasingly used for convenience and safety, but bleeding risk must be assessed with tools like the (Hypertension, Abnormal renal/liver function, Stroke, Bleeding, Labile INR, Elderly, Drugs/alcohol) HAS-BLED score. Notably, anticoagulation recommendations differ across various PH groups. For patients in Group 1 (pulmonary arterial hypertension, PAH), recent studies indicate that anticoagulation is considered an optional therapy in the absence of a definitive history of thromboembolism, necessitating careful consideration. In contrast, for patients in Group 4 (CTEPH), given that venous thromboembolism constitutes the core etiology, lifelong anticoagulation is recommended irrespective of the presence of AF, unless contraindications exist [79,80]. In summary, anticoagulation management in patients with PH should be individualized by clinicians based on the specific subgroup to achieve an optimal risk–benefit balance.

### 4.3 Interventional and Device Therapy

#### 4.3.1 Catheter Ablation

Catheter ablation is a key treatment for PH patients with specific arrhythmias, like typical AFL (dependent on the cavotricuspid isthmus). It shows an acute success rate up to 90% but a 3-year recurrence rate of 78.3%. Despite this high recurrence, catheter ablation remains the first-line strategy for typical AFL [81,82]. AF ablation in PH patients is more challenging due to significant atrial remodeling, fibrosis, and matrix abnormalities, which complicate AF maintenance and result in higher recurrence rates than in the general population, despite pulmonary vein isolation (PVI) being the standard approach [83]. Studies indicate that a PAP ≥35 mmHg independently predicts recurrence after paroxysmal AF ablation [84]. Radiofrequency catheter ablation appears short-term safe in PH patients, with no evidence of directly triggering acute right heart failure or a PH crisis [85,86]; however, recurrence still exceeds that in non-PH populations [84,87]; therefore, clinicians are inclined to employ more extensive atrial substrate modification strategies in clinical practice. However, there is currently a paucity of large-scale meta-analyses or systematic reviews specifically targeting PH patients to validate the efficacy of this therapeutic approach, and the existing evidence remains to be consolidated. Notably, even within the general AF population, multiple RCTs have compared PVI alone with PVI plus adjunctive substrate modification—including linear ablation, posterior wall isolation, complex fractionated electrogram ablation, MRI-guided fibrosis ablation, and low-voltage zone ablation—yet their conclusions are inconsistent [88], further complicating the individualization of treatment strategies for PH patients.

Although the overall risk of catheter ablation is modest, independent predictors of elevated risk include VT and AF ablation (vs. other substrates), advanced age, female sex, multimorbidity, and low procedural volume. Principal complications are cardiac tamponade, major hemorrhage, stroke, and vascular injury [89]. Limited systematic data are available regarding the specific risks of invasive catheter ablation for arrhythmias in patients with PH; however, this population is likely to face more complex periprocedural challenges compared with the general population. PH patients frequently exhibit right heart remodeling, characterized by marked right atrial enlargement, and some may present with coronary sinus dilation, particularly in the context of congenital venous anomalies, which can increase the technical difficulty of venous access and catheter manipulation. Right atrial enlargement may also predispose certain atrial fibrillation subtypes to a higher risk of post‑ablation recurrence [90]. Vasovagal reflexes constitute an important intraprocedural complication [91]. Patients with PH may present with right ventricular dysfunction [2], which limits the ventricle’s compensatory capacity in response to vagal stimuli; consequently, the occurrence of severe bradycardia or asystole can lead to hemodynamic instability. The relationship between individual patient sensitivity to sedatives/anesthetics and post‑ablation complications remains inconclusive. In light of the frequently elevated pulmonary vascular resistance—and the potential for right‑to‑left shunting in advanced or severe PH—excessive sedation may suppress respiratory drive and precipitate acute right heart failure, whereas inadequate sedation may lead to patient agitation and procedural failure. Consequently, anesthetic strategies must be carefully individualized to balance these competing risks. Moreover, some PH patients (e.g., those with CTEPH) experience progressive oxygen desaturation even under mild sedation due to ventilation‑perfusion mismatch [92], which imposes additional demands on maintaining intraoperative circulatory stability. Although large‑scale prospective studies directly correlating SpO_2_ monitoring with catheter ablation outcomes are lacking, continuous pulse oximetry remains a clinically prudent measure in this high‑risk population.

#### 4.3.2 Pulmonary Artery Denervation

Pulmonary artery denervation (PADN) is an investigational therapy that ablates sympathetic ganglia in the pulmonary artery to block conduction and reduce pressure. Studies show safety and potential benefits in symptoms, hemodynamics, and imaging, but clinical application is limited due to a lack of large-scale RCT evidence and unclear efficacy differences among techniques like radiofrequency ablation and high-intensity focused ultrasound (HIFU) [39,93]. PADN shows potential to improve the 6MWD and reduce mPAP compared to traditional therapies as research advances [94,95].

### 4.4 Surgical and End-of-Life Treatments

Advanced treatment options should be considered for end-stage PH patients failing drug and interventional treatments with worsening conditions.

#### 4.4.1 Atrial Septostomy/Reverse Potts Shunt

Atrial septostomy is a palliative interventional procedure that uses a balloon to create an atrial septal defect, enabling right-to-left shunting to reduce right ventricular overload, primarily as transitional therapy for patients awaiting lung transplantation. It is not intended for the treatment of arrhythmia [96]. Reverse Potts shunting (RPS) creates a right-to-left shunt between the pulmonary artery and descending aorta, effectively treating certain pediatric PH cases, but requires strict indication adherence and risk-benefit analysis [97].

#### 4.4.2 Mechanical Circulatory Support (MCS)

For patients with refractory arrhythmias causing acute right heart failure or cardiogenic shock, venoarterial extracorporeal membrane oxygenation (VA-ECMO) can be used as temporary circulatory support in those who are lung-transplant candidates or have a reversible cause (e.g., new-onset arrhythmia or infection), serving to stabilize vital signs and enable subsequent treatment [98]. VA-ECMO is not indicated for the direct management of arrhythmias. Right ventricular assist devices (RVADs) are reserved for highly selected cases, given the limited evidence of long-term success and inherent pathophysiological risks [99].

#### 4.4.3 Lung Transplantation

Lung transplantation is key for end-stage PH, reducing PVR and improving right heart function [100], and extends survival to 61% at 5 years, requiring strict indication adherence and comprehensive risk-benefit analysis [101].

### 4.5 Emerging and Advanced Treatment Methods

#### 4.5.1 Targeting Myocardial Remodeling and the Electrophysiological Matrix

Cx43-Targeted Therapies: Cx43 is crucial for electrical impulse conduction in atrial cells. In PH-induced right atrial pressure overload, Cx43 expression is downregulated and lateralized, disrupting electrical coupling, forming slow conduction zones, and promoting reentrant arrhythmias like AF. Drugs such as Gap26 peptide show potential to reduce AF in animal models and are transitioning from preclinical to early clinical trials, but lack large-scale animal or human trial evidence [20]. Anti-inflammatory and antifibrotic treatments target inflammation and fibrosis, crucial for right heart remodeling and arrhythmia substrate formation in PH patients [20]. The NOD-like receptor protein 3 (NLRP3) inflammasome is a key platform for inflammatory responses, releasing factors like IL-1β when activated. Melatonin and other NLRP3 inhibitors are effective in animal models, reducing pulmonary vascular remodeling through this pathway [102], and may reduce arrhythmia susceptibility. Additionally, Bone Morphogenetic Protein (BMP)/TGF-β pathway drugs are in development. Sotatercept, the first BMP/TGF-β pathway regulator, uses ligand trap technology to restore signal balance. Phase III trials confirmed that it reduces PVR and improves 6MWD [103]. Preclinical investigations and initial clinical trials have evidenced favorable outcomes in pulmonary vascular remodeling; nonetheless, the precise effects on arrhythmias associated with PH have not been established.

#### 4.5.2 Gene and Cell Therapy

Gene Therapy: 70%–80% of hereditary PH is caused by *BMPR2* mutations [104]. Adenovirus-mediated switch-independent 3A (*SIN3A*) gene introduction corrects *BMPR2* epigenetic silencing, restores expression, and improves PH. Currently in preclinical trials, it must address translational medicine issues to advance PH-related arrhythmia treatment [105]. miRNAs regulate gene expression in PH pathophysiology. Studies show that multiple miRNAs and their upstream regulators (e.g., transcription factors, competing endogenous RNA (ceRNA) networks) collectively modulate pulmonary vascular remodeling and right ventricular dysfunction [23]. For example, miR-204 supplementation inhibits Src–STAT3–NFAT signaling, inhibits PASMC proliferation and promotes apoptosis, reduces pulmonary artery pressure, improves vascular remodeling, and upregulates *BMPR2* expression to repair the pathway [22]. Cell therapy with mesenchymal stem cells (MSCs) provides a new treatment strategy for PH-related arrhythmias. MSCs are a potential PH treatment; animal studies show transplantation alleviates symptoms, and conditioned medium reverses hypoxia-induced endothelial-to-mesenchymal transition (EndMT), suggesting paracrine mechanisms. Intravenous injection is safe and significantly reduces PVR [106,107]. MSCs improve pulmonary vascular lesions, suppressing myocardial fibrosis and indirectly reducing arrhythmias. However, its efficacy in preventing arrhythmias among patients with PH remains merely conjectural, and empirical clinical validation has yet to be established. The translation of miRNA research findings into clinical applications is confronted with multiple challenges. First, technological heterogeneity and lack of standardization constitute the primary obstacle to the reproducibility of miRNA biomarkers. Significant variations exist across studies in terms of sample types (e.g., plasma versus serum), RNA extraction methodologies, detection platforms (such as quantitative reverse transcription polymerase chain reaction (qRT-PCR), microarray, and next-generation sequencing), and data normalization strategies (including the selection of reference genes like U6 and cel-miR-39). These discrepancies hinder direct comparison and integration of results from different investigations. Inter-laboratory operational differences (such as variations in reagent batches and personnel expertise) further exacerbate this issue. Although guidelines from the External RNA Controls Consortium (ERCC) and the Minimum Information for Publication of Quantitative Real-Time PCR Experiments (MIQE) provide a framework for standardization, their adoption and application in the field of PH remain inadequate [108]. Second, the high heterogeneity of PH and the subtype-specific expression profiles of miRNAs have been extensively confirmed in numerous studies. However, the principal obstacle to the clinical translation of miRNA biomarkers resides in technical standardization, encompassing sample processing, platform variability, and normalization strategies, as well as the validation of specificity. Future investigations should prioritize prospective validation of multi-miRNA combination models in large-scale cohorts that encompass populations with diverse subtypes and comorbidities [108]. Third, disruption of the clinical validation chain remains a critical limitation. Nearly all existing studies on miRNA biomarkers are retrospective case-control studies with limited sample sizes. There is a critical shortage of prospective, large-sample, multicenter longitudinal cohort studies designed to validate the clinical utility of these biomarkers for disease risk stratification, early diagnosis, prognosis prediction, or treatment response assessment [109]. Additionally, comorbidities (e.g., chronic obstructive pulmonary disease [COPD]) and miRNAs released due to red blood cell lysis (such as miR-451a) may confound disease-specific signals, thereby increasing the complexity of clinical application [108]. Fourth, insufficient efficiency and specificity of delivery systems represent a major technical barrier. Following systemic administration, therapeutic miRNA molecules or vectors are prone to rapid clearance by the liver, resulting in low bioavailability at the target pulmonary vasculature. Concurrently, this may induce systemic off-target effects. The development of vectors capable of efficiently and specifically delivering miRNAs to pulmonary vascular cells—particularly pulmonary artery endothelial cells, the primary target cell type for hypoxic pulmonary hypertension (HPH) intervention—remains an unresolved key technical challenge [108]. Fifth, off-target effects and unknown long-term safety raise significant concerns. The pleiotropy of miRNAs implies that a single miRNA can regulate hundreds of target genes, leading to complex regulatory networks. This may precipitate unpredictable off-target effects, interfering with normal physiological pathways and raising safety concerns. For chronic conditions like PH that require long-term treatment, the long-term safety of miRNA therapies remains unknown, as no HPH-specific miRNA therapy has yet entered late-stage clinical trials, and rigorous preclinical safety profiling and long-term biocompatibility data are still needed [108]. Finally, clinical development strategies and efficacy evaluation present substantial dilemmas. The high heterogeneity of PH subtypes challenges clinical trial design, rendering patient selection and the definition of clinical endpoints particularly arduous. The absence of validated confirmatory biomarkers for patient stratification and treatment monitoring impedes the objective assessment of miRNA therapy’s effects on vascular structure improvement in the short term. Furthermore, the existing evidence supporting miRNA therapy predominantly originates from preclinical studies with limited sample sizes, and to date, no miRNA-based therapeutics targeting HPH have progressed to phase II or III clinical trials. Consequently, prospective, large-scale, multicenter longitudinal studies are critically necessary to validate the long-term safety and clinical efficacy of these interventions [108].

## 5. Conclusion

PH-related arrhythmias critically influence disease progression and survival. This review consolidates evidence on the pathophysiological network linking hemodynamic, structural, metabolic, electrophysiological, and inflammatory pathways. Despite recent advances, key gaps remain: the absence of Phase II/III trials with arrhythmias as a primary endpoint in PH, overreliance on animal models, limited static end-stage mechanistic evidence, and a lack of unified guidelines—although individualized management is advised. Future efforts should prioritize translational studies with early-phase mechanistic trials, humanized disease models for drug screening and discovery, and AI-driven risk stratification enabling dynamic prediction and personalized therapy to improve PH outcomes.
